# New options for cilostazol-based dual antiplatelet therapy for ischaemic stroke prevention in East Asian populations: a systematic review and meta-analysis

**DOI:** 10.3389/fphar.2025.1533674

**Published:** 2025-02-19

**Authors:** Xiaorui Wang, Yixin Wang, Qiang Zheng, Pengwei Li, Jingli Yin, Miaomiao Wang, Liangyu Zou, Jie Wang, Jialin Pan, Lei Qin, Song Luo, Lijuan Yang

**Affiliations:** ^1^ Department of Neurology, The First Affiliated Hospital of Bengbu Medical University, Bengbu, China; ^2^ Department of Pediatrics, The First Affiliated Hospital of Bengbu Medical University, Bengbu, China; ^3^ Department of Neurology, Yichuan People’s Hospital, Luoyang, China; ^4^ Department of Emergency, Yichuan People’s Hospital, Luoyang, China; ^5^ Department of Neurology, Shenzhen People’s Hospital, The Second Clinical Medical College, Jinan University, Shenzhen, China; ^6^ International Medical Center (Department of Geriatric Medicine), Shenzhen University General Hospital, Shenzhen, China; ^7^ Department of Internal Medicine, Second People’s Hospital, Longgang, Shenzhen, China; ^8^ Department of Radiology, The First Affiliated Hospital of Bengbu Medical University, Bengbu, China

**Keywords:** ischaemic stroke, cilostazol, dual antiplatelet therapy, meta-analysis, systematic review

## Abstract

**Background and purpose:**

The objective of this study is to systematically review the efficacy and safety of cilostazol-based dual antiplatelet therapy (DAPT) in patients with stroke.

**Methods:**

Two reviewers conducted a comprehensive search of eligible studies published in PubMed, Medline, the Cochrane Library, Embase, and four Chinese databases from their establishment to 31 July 2024. The review was registered (CRD42024559047).

**Results:**

This study included a total of 4,473 subjects from 11 studies. The results indicated that, when compared to aspirin/clopidogrel single antiplatelet therapy (SAPT), cilostazol-based DAPT was associated with lower ischemic stroke (RR = 0.54, 95% CI 0.38–0.75, *P* = 0.0003) and any stroke recurrence (RR = 0.52, 95% CI 0.31–0.86, *P* = 0.01). Furthermore, the incidence of general adverse events was higher in the cilostazol-based DAPT (RR = 1.93, 95% CI 1.16–3.21, P = 0.01), while no statistically significant difference was observed between the two groups with regard to serious adverse events. The subgroup analysis of follow-up time revealed that the cilostazol-based DAPT regimen demonstrated superior efficacy in reducing the incidence of ischemic stroke recurrence (RR = 0.51; 95% CI 0.36–0.73; P = 0.0002) and any stroke recurrence (RR = 0.49; 95% CI 0.35–0.67; P < 0.0001) in the long-term (>3 months) versus the short-term (≤3 months) group. Furthermore, the cilostazol-based DAPT regimen did not increase the risk of serious adverse events.

**Conclusion:**

DAPT combined with cilostazol and aspirin or clopidogrel was superior to aspirin or clopidogrel alone, did not increase serious adverse events, and was more effective for long-term (>3 months) prophylaxis.

**Systematic Review Registration:**

https://www.crd.york.ac.uk/PROSPERO/, identifier CRD42024559047

## Introduction

Patients presenting with a minor ischemic stroke (IS) or transient ischemic attack (TIA) exhibit a markedly elevated risk of recurrent stroke (Amarenco et al., 2018). In China, the recurrence rates of stroke within 3 and 12 months are 3.6% and 5.6%, respectively, and the mortality rates are 4.2% and 8.5%, respectively (Tu et al., 2023). Identifying secondary prevention strategies for this high-risk group is essential to reduce morbidity and mortality. The administration of dual antiplatelet therapy (DAPT) comprising aspirin and clopidogrel within 24 h of symptom onset and for a period of 3 weeks has been demonstrated to reduce the recurrence of stroke in select patients presenting with high-risk transient ischemic attack (TIA) and minor strokes (Mendelson and Prabhakaran, 2021). However, this therapeutic approach is associated with an increased risk of causing moderate-to-severe hemorrhage (Gao et al., 2023). In light of the advent of genetic testing and the growing prevalence of aspirin/clopidogrel resistance, the pursuit of safer and more efficacious alternatives has become a pressing concern in contemporary medical research. Cilostazol is a selective inhibitor of phosphodiesterase 3, which not only inhibits platelet aggregation but also contributes to vasodilation and inhibits vascular smooth muscle cell proliferation (Zheng et al., 2019). The American Cardiovascular Society recommends cilostazol as a first-line pharmacotherapy for the management of intermittent claudication resulting from peripheral vascular disease (Gornik et al., 2024). Studies conducted by CSPS have shown that combining cilostazol with aspirin or clopidogrel can decrease the occurrence of ischemic events in high-risk ischemic stroke patients without raising the risk of bleeding (Toyoda et al., 2022). Currently, cilostazol is recognized as a second-line medication for preventing secondary stroke in China, South Korea, and other East Asian countries (Yongjun et al., 2022; Park et al., 2022), but it is not widely accepted in most countries. Thus, we have conducted a systematic review and meta-analysis to assess the effectiveness and safety of cilostazol-based DAPT in preventing stroke recurrence among Asian populations. Our aim is to provide trustworthy evidence for clinical applications.

## Methods

This paper reports in compliance with the Preferred Reporting Items for Systematic Reviews and Meta Analyses statement (PRISMA).

### Search strategy

A comprehensive search was conducted across eight databases, including PubMed, Medline, Cochrane Library, Embase, four Chinese databases (China National Knowledge Infrastructure (CNKI), Chinese Biomedical Literature database (CBM), Wanfang Digital Periodicals (Wanfang) and Chinese Science and Technology Periodicals (VIP) database), to identify randomized controlled trials (RCTs), non-RCTs, and cohort studies related to the study. The search spanned from the time of construction to 31 July 2024. Furthermore, references to the included literature were examined to identify additional sources of relevant information. A combination of subject terms and free terms was used to search for literature related to ischemic stroke. The following search terms were employed: The search terms used were “Ischemic Attack, Transient,” “Transient Ischemic Attack”, “Ischemic Stroke” and “Acute Ischemic Stroke”. The search terms related to Cilostazol are “Cilostazol”, “Pletal”, “OPC 13013” or “OPC-13013.” Please refer to the Supplementary Table S1 for detailed search strategies.

### Study selection

The included studies met the following criteria: (1) They were randomized controlled trials, non-randomized concurrent controlled trials, or cohort studies; (2) The study subjects were patients with IS or TIA; (3) Single antiplatelet drug therapy (SAPT) (aspirin/clopidogrel) was administered to the control group, and DAPT containing cilostazol in combination with aspirin/clopidogrel was administered to the intervention group. (4) Outcome Indicators: Effectiveness indicators include ischemic stroke recurrence (Thiraworawong and Pathonsmith, 2024), any stroke recurrence [ischemic stroke recurrence, hemorrhagic stroke, neurological deterioration (Aoki et al., 2019)], while safety indicators include serious adverse events (intracranial hemorrhage, cardiovascular events, death, Bleeding events) and general adverse events (other than bleeding events, non-fatal events, include headache, dizziness, palpitations, skin rash, gastrointestinal symptoms, and so forth).

### Data collection and quality assessment

The two researchers conducted independent reviews of the literature, initially screening and evaluating the titles and abstracts to exclude those that clearly did not meet the inclusion criteria. They then re-screened the remaining literature to identify the full texts. Duplicate publications, data that could not be extracted, full texts that could not be retrieved, or conference literature were excluded. The literature identified for inclusion in the final meta-analysis was confirmed and the data extracted. In the event of a discrepancy, a third researcher was consulted to facilitate a discussion and achieve a consensus.

The content of data extraction includes the following: (1) Basic information of the included studies, such as first author, publication year, and study location; (2) Baseline characteristics of the study subjects, including sample size, age, and gender; (3) Specific details of the intervention measures and follow-up time; (4) Outcome indicators of interest in this study and result measurement data; and (5) Key elements of bias risk assessment.

Two researchers evaluated the risk of bias in randomized controlled trials (RCTs) using the RCT bias risk assessment tool from the Cochrane Handbook. Non-RCTs were assessed for bias risk using the MINOR scale, while cohort studies were evaluated for bias risk using the Newcastle-Ottawa Scale (NOS).

### Statistical analysis

Meta-analysis was performed using RevMan5.4 software. For counting data, the relative risk ratio (RR) was used as the effect index, and 95% confidence interval (CI) was calculated. Heterogeneity was analyzed by *I* ^*2*^ test, if *P* < 0.10 and *I* ^*2*^ > 50%, it was considered that there was significant heterogeneity among the studies. Random effects model was used for meta-analysis, and sensitivity analysis or subgroup analysis was performed; otherwise, fixed effects model was selected for meta-analysis.

## Results

### Study selection

**FIGURE 1:**
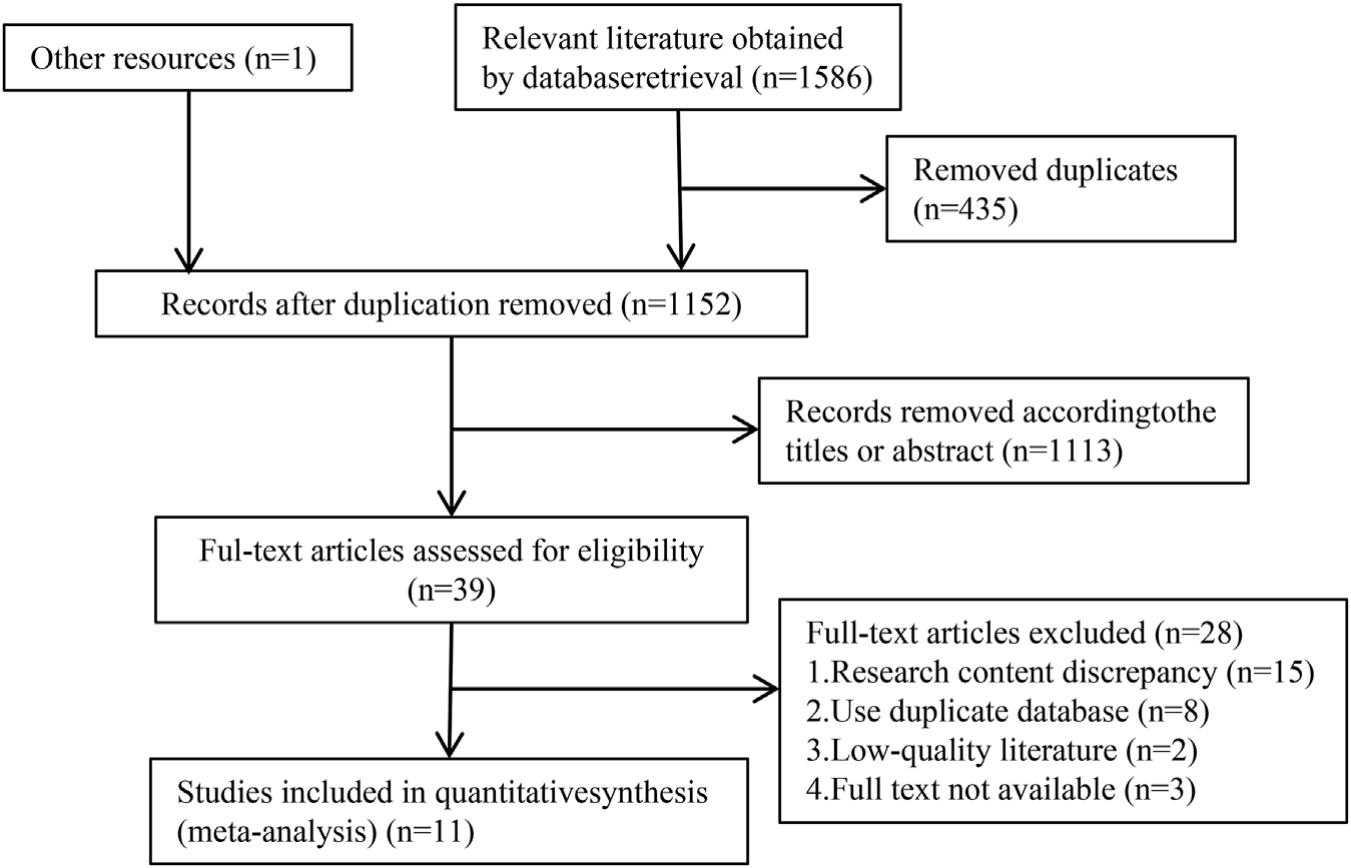
Study flowchart.

The process of literature search for meta-analysis is illustrated in Figure 1. A total of 1,587 relevant studies were identified by conducting searches in 8 databases, namely PubMed (103), Medline (262), Cochrane Library (91), Embase (961), CNKI (46), Wanfang (47), VIP (33), CBM (43), and other sources (1). After removing 435 duplicates, the titles and abstracts of 1,152 documents were screened to exclude 1,113 documents that did not meet the inclusion criteria. Subsequently, 39 full-text documents were reviewed, which led to the exclusion of 28 documents. Finally, 11 studies that met the inclusion criteria for meta-analysis were included.

### Study characteristics

The characteristics of the study are presented in Table 1. A total of 11 studies were included, with 4,473 participants, of which 10 were RCTs and 1 was a cohort study (Thiraworawong and Pathonsmith, 2024). All studies were conducted in Asia, with two from China, 1 from Thailand, 3 from South Korea, and 5 from Japan. The participants were primarily non-cardioembolic ischemic stroke patients, with a minimum age of 18 and a maximum age of 80, with males accounting for approximately 66.4% of the participants. Six studies had a control group receiving single aspirin therapy, while three studies had a control group receiving aspirin combined with placebo therapy. The follow-up period ranged from 14 days to 3.5 years.

**TABLE 1:** Characteristics of included studies.

Study	Country	Treatment onset	Sample size,n	Age,y (I/C)	Mela,n (I/C)	Dual Group	Monotherapy	Follow-up time
(ADS) Aoki et al. (2019)	Japan	Noncardioembolic stroke within 48 h	1,201	69/69	398/398	Cilostazol (200 mg/day)+aspirin (80–200 mg/day)	Aspirin (80–200 mg/day)	90 days
(ECLIPse) Han et al. (2013)	Korea	Lacunar infarction within 7 days	203	64.63/65.48	74/78	Cilostazol (100 mg, twice daily) + aspirin (100 mg/day)	Aspirin (100 mg/day) +placebo	90 days
(CSPS.com) Toyoda et al. (2019)	Japan	Noncardioembolic ischemic stroke within 8–180 days	1879	NA/NA	NA/NA	Cilostazol (100 mg, twice daily) +aspirin (81 or 100 mg/day)/clopidogrel (50 or 75 mg/day)	Aspirin (81 or 100 mg/day)/clopidogrel (50 or 75 mg/day)	0.5–3.5 years
Kwon et al. (2005)	Korea	Ischemic stroke within 2 weeks	135	62.18/62.54	41/41	Cilostazol (100 mg, twice daily) +aspirin (100 mg/day)	Aspirin (100 mg/day)+ placebo	6 months
Lee et al. (2010)	Korea	Ischaemic stroke and had received aspirin 100 mg a day for at least 2 weeks	244	61.2/62.8	89/78	Cilostazol (100 mg, twice daily) +aspirin (100 mg/day)	Aspirin (100 mg/day)+ placebo	4 weeks
Nakamura et al. (2012)	Japan	Noncardioembolic ischemic stroke within 48 h	76	66/67	29/27	Cilostazol (100 mg, twice daily) +aspirin (300 mg/day)	Aspirin (300 mg/day)	6 months
Thiraworawong and Pathonsmith (2024)	Thailand	Transient ischemic attack or acute ischemic stroke and asymptomatic atherosclerotic carotid artery stenosis	314	64/65	89/92	Cilostazol (200 mg/day) + aspirin (81–100 mg/day)/clopidogrel (75 mg/day)	Aspirin (81–325 mg/day)/clopidogrel (75 mg/day)	1 year
(CATHARSIS) Uchiyama et al. (2015)	Japan	Ischemic stroke within 2 weeks–6 months	163	68.3/68.3	64/43	Cilostazol (200 mg/day)+aspirin (100 mg/day)	Aspirin (100 mg/day)	2 years
Zhang and Chen (2019)	China	Noncardioembolic ischemic stroke within 48 h	60	64.1/63.4	17/19	Cilostazol (100 mg, twice daily) +aspirin (100 mg/day)	Aspirin (100 mg/day)	6 months
Cao and Wang (2015)	China	Noncardioembolic ischemic stroke within 48 h	174	64.8/66.0	67/62	Cilostazol (100 mg, twice daily) +aspirin (300 mg/day)	Aspirin (300 mg/day)	14 days
Ohnuki et al. (2017)	Japan	Noncardioembolic ischemic stroke within 1 week	24	60.5/63.6	9/8	Cilostazol (200 mg/day) +aspirin (100 mg/day)	Aspirin (100 mg/day)	4 weeks

I, Intervention group; C, Control group.

### Methodological quality assessment

The majority of the included studies exhibited minimal risk of bias across the seven domains examined in the meta-analysis (Supplementary Figure S1). Thiraworawong (Thiraworawong and Pathonsmith, 2024) was rated as high-quality literature using the NOS scale, with the exception of the entry “Demonstration that outcome of interest was not present at the start of the study,” which was rated 8 out of 10, indicating high-quality literature.

### Effectiveness indicators

**FIGURE 2:**
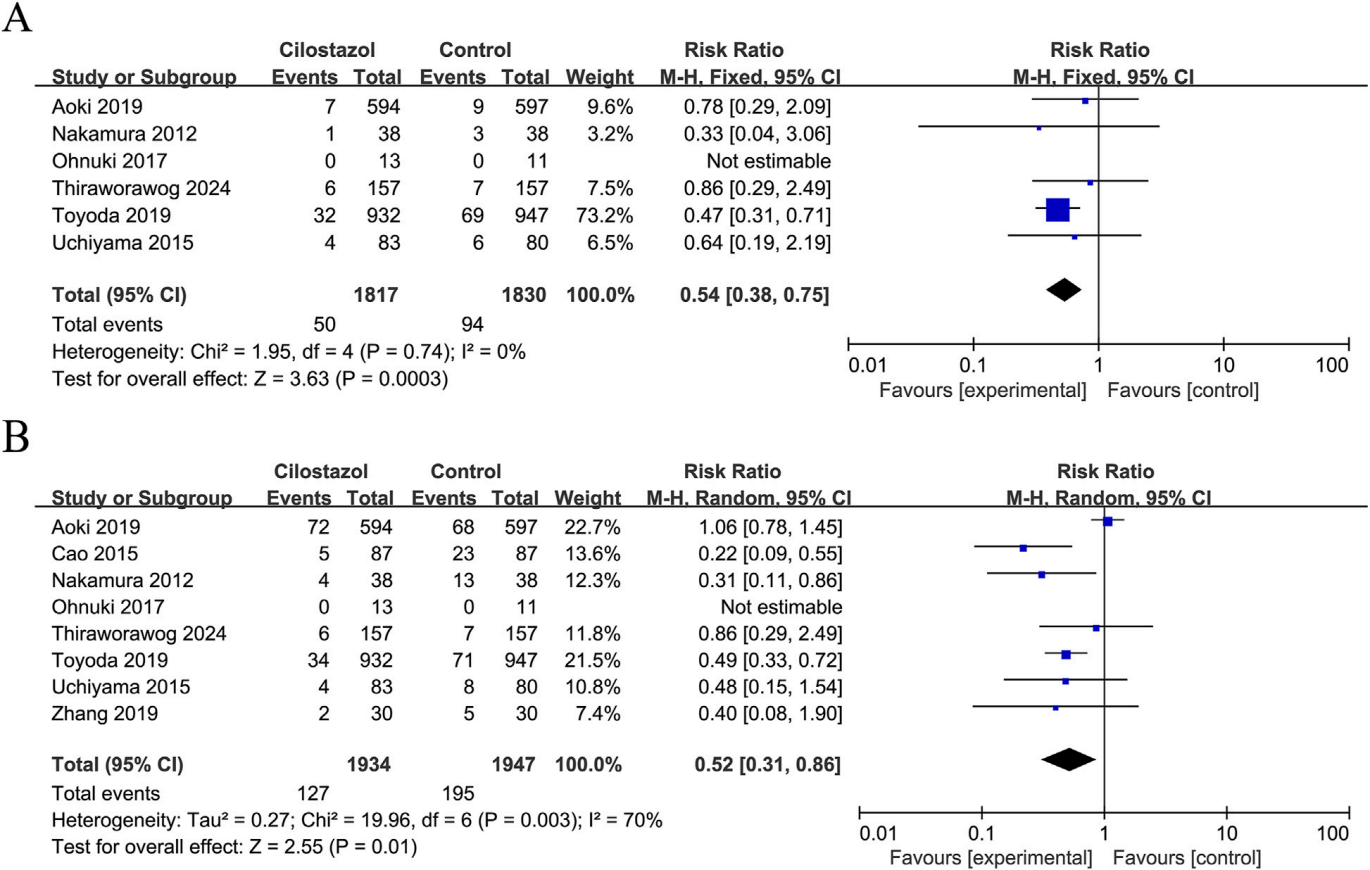
Forest plot of effectiveness indicators. **(A)** Ischemic stroke recurrence; **(B)** Any stroke recurrence.

Six studies reported ischaemic stroke recurrence and included a total of 3,647 patients (Figure 2A). The results showed that cilostazol-based DAPT significantly reduced ischaemic stroke recurrence compared with SAPT (RR = 0.54; 95% CI 0.38–0.75; *P* = 0.0003) with no heterogeneity (*I* ^*2*^ = 0%; *P* = 0.74).

Eight studies reported data on any stroke recurrence, including a total of 3,881 patients (Figure 2B). The results demonstrated that cilostazol-based DAPT was associated with a significantly reduced incidence of any stroke recurrence in comparison to SAPT (RR = 0.52; 95% CI 0.31–0.86; *P* = 0.01). Due to inter-study heterogeneity (*I* ^*2*^ = 70%; *P* = 0.003), a sensitivity analysis was conducted using a case-by-case exclusion method. Following the removal of the study by Aoki et al. (2019), the heterogeneity was no longer significant (I^2^ = 0%, *P* = 0.48), and the combined results remained largely unchanged (RR = 0.44; 95% CI 0.32–0.59; *P* < 0.00001) (Supplementary Figure S2).

### Safety indicators

#### Serious adverse events

**FIGURE 3:**
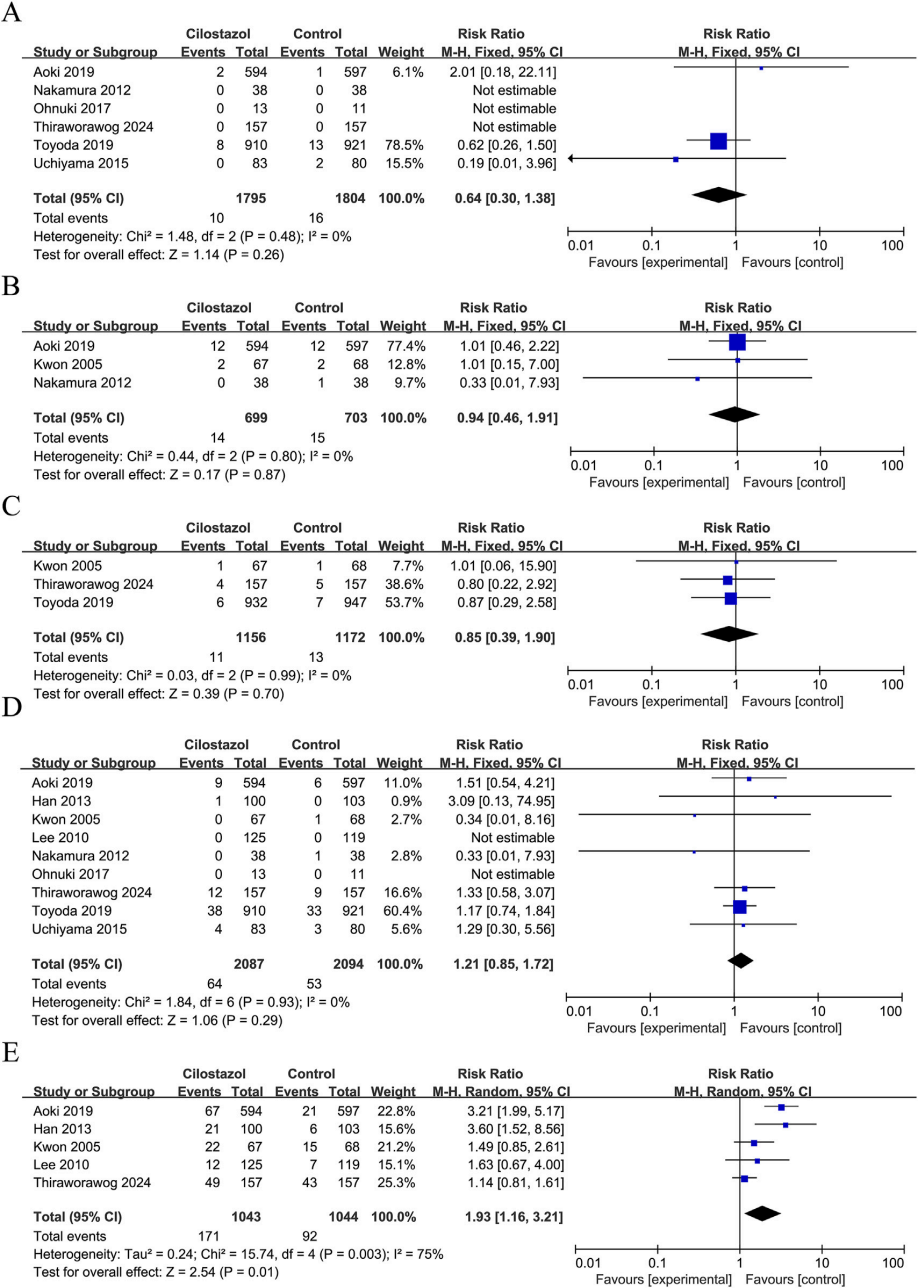
Forest plot of safety indicators. **(A)** Intracranial hemorrhage; **(B)** Cardiovascular events; **(C)** Death; **(D)** Bleeding events; **(E)** General adverse events.

A total of six studies reported intracranial haemorrhage, three studies reported cardiovascular events, three studies reported death, and nine studies reported bleeding events. The study findings indicate that there were no statistically significant differences observed between cilostazol-based DAPT and SAPT in terms of intracranial hemorrhage (RR = 0.64; 95% CI 0.30–1.38; *P* = 0.26), cardiovascular events (RR = 0.94; 95% CI 0.46–1.91; *P* = 0.87), death (RR = 0.85; 95% CI 0.39–1.90; *P* = 0.70), and bleeding events (RR = 1.21; 95% CI 0.85–1.72; *P* = 0.29). Furthermore, none of the heterogeneity was significant (*I* ^*2*^ = 0%, *P* > 0.05) (Figure 3).

#### General adverse event

A total of five studies reported the occurrence of general adverse events, with a total of 2,087 patients enrolled (Figure 3). As demonstrated by the results, there was considerable heterogeneity (*I* ^*2*^ = 75%, *P* = 0.003) in terms of the increased incidence of general adverse events with cilostazol-based DAPT compared to SAPT (RR = 1.93; 95% CI 1.16–3.21; *P* = 0.01) (Figure 3). A sensitivity analysis was conducted using a case-by-case exclusion method, following the removal of Thiraworawong and Pathonsmith (2024), the heterogeneity was no longer significant (*I* ^*2*^ = 49%, *P* = 0.12), and the combined results remained largely unchanged (RR = 2.50; 95% CI 1.83–3.42; *P* < 0.00001) (Supplementary Figure S3).

### Subgroup analysis

In this study, subgroups were analyzed based on the duration of follow-up, with a cutoff of 3 months. The short-term group (≤3 months) consisted of 5 studies with a total of 1,846 participants, while the long-term group (>3 months) consisted of 6 studies with a total of 2,627 participants (Table 2). The short-term group did not demonstrate a significant preventive effect on recurrent ischemic stroke (RR = 0.78; 95% CI 0.29–2.09; *P* = 0.62) or any stroke recurrence (RR = 0.51; 95% CI 0.11–2.44; *P* = 0.40). In contrast, the long-term group exhibited a reduced risk of recurrent ischemic stroke (RR = 0.51; 95% CI 0.36–0.73; *P* = 0.0002) and any stroke recurrence (RR = 0.49; 95% CI 0.35–0.67; *P* < 0.0001). However, heterogeneity was observed in any stroke recurrence (*I* ^*2*^ = 70%, *P* = 0.003). The incidence of general adverse events was higher in the short-term group (RR = 2.91; 95% CI 1.99–4.25; *P* < 0.00001). However, there was no significant difference in the long-term group (RR = 1.23; 95% CI 0.91–1.64; *P* = 0.17), although there was heterogeneity (*I* ^*2*^ = 75%, *P* = 0.003). There was no significant difference observed in the long-term versus short-term safety regarding intracranial hemorrhage, cardiovascular events, and bleeding events among serious adverse events (Supplementary Figures S4–S9). However, the analysis of deaths was not conducted due to insufficient data.

**TABLE 2:** Subgroup analysis (Based on follow-up time).

Outcome	Short term					Long term					Total term				
	RR (95%CI)	N	Weight (%)	*I* ^2^ (%)	*P*	RR (95%CI)	N	Weight (%)	*I* ^2^ (%)	*P*	RR (95%CI)	N	Weight (%)	*I* ^2^ (%)	*P*
Ischaemic stroke recurrence	0.78 (0.29,2.09)	2	9.6	NA	NA	0.51 (0.36,0.73)	4	90.4	0	0.72	0.54 (0.38,0.75)	6	100	0	0.740
Any stroke recurrence	0.51 (0.11,2.44)	3	36.3	90	0.001	0.49 (0.36,0.68)	5	63.7	0	0.75	0.52 (0.31–0.86)	8	100	70	0.003
Intracranial hemorrhage	2.01 (0.18,22.11)	2	6.1	NA	NA	0.55 (0.24,1.27)	4	93.9	0	0.46	0.64 (0.30,1.38)	6	100	0	0.480
Cardiovascular events	1.01 (0.46,2.22)	1	77.4	NA	NA	0.72 (0.15,3.57)	2	22.6	0	0.56	0.94 (0.46,1.91)	3	100	0	0.800
Death	NA	NA	NA	NA	NA	NA	NA	NA	NA	NA	NA	NA	NA	NA	NA
Bleeding events	1.63 (0.62,4.30)	4	11.9	0	0.670	1.15 (0.79,1.68)	5	88.1	0	0.86	1.21 (0.85,1.72)	9	100	0	0.930
General adverse events	2.91 (1.99,4.25)	3	53.6	0	0.370	1.23 (0.91,1.64)	2	46.4	0	0.43	1.93 (1.16,3.21)	5	100	75	0.003

RR, relative risk ratio; CI, confidence interval.

## Discussion

The present study demonstrated that DAPT with cilostazol in combination with aspirin or clopidogrel resulted in a notable reduction in both ischemic and any stroke recurrence compared to single aspirin or clopidogrel. Furthermore, there was no significant difference in the incidence of serious adverse events. In the long-term cilostazol-based DAPT group (>3 months), a further reduction in both ischemic and any stroke recurrence was observed compared to the short-term group (≤3 months). Additionally, there was no increase in the risk of serious adverse events. The long-term treatment with cilostazol-based DAPT demonstrated a more pronounced benefit.

The results of the combination therapy with cilostazol are promising. The difference in safety and efficacy between cilostazol-based DAPT and aspirin monotherapy was not statistically significant (Tan et al., 2021; Tan et al., 2015; McHutchison et al., 2020). A meta-analysis was not conducted to evaluate the efficacy of cilostazol-based DAPT versus clopidogrel monotherapy due to the limited number of studies that made these comparisons. However, the available evidence suggests that the combination of cilostazol and clopidogrel may reduce the incidence of secondary ischemic strokes without increasing the risk of hemorrhage compared with clopidogrel monotherapy (Toyoda et al., 2019). In this study, we found that cilostazol-based DAPT was superior to SAPT in preventing stroke recurrence and did not increase the risk of bleeding. Exploring which regimen is superior, cilostazol in combination with aspirin or clopidogrel, will be the next step of research. In a separate meta-analysis of cilostazol monotherapy and combination therapy, cilostazol drugs demonstrated greater efficacy in reducing recurrent and ischemic stroke when administered in combination. Conversely, they exhibited superior benefits in hemorrhagic stroke when utilized as monotherapy (Kim et al., 2019). Moreover, a study was conducted to compare the efficacy of cilostazol-based DAPT with that of aspirin combined with clopidogrel in the progression of symptomatic intracranial atherosclerotic stenosis (ICAS) associated with stroke recurrence. The study revealed that the two combined antiplatelet therapies demonstrated no significant difference in preventing the progression of ICAS and the incidence of new ischemic lesions and hemorrhage in patients with symptomatic ICAS. However, the incidence of headaches was higher in the cilostazol group. The incidence of headaches was 26.7% in the cilostazol group and 15.1% in the clopidogrel group (Kwon et al., 2011). When patients display aspirin/clopidogrel resistance, switching to cilostazol may prove to be an efficacious therapeutic alternative, while simultaneously avoiding the increased incidence of serious adverse events. A meta-analysis of the prophylactic role of cilostazol alone versus aspirin alone in secondary stroke revealed that cilostazol was associated with a lower incidence of stroke and a significantly lower rate of hemorrhage. However, it was also associated with a higher incidence of headache and dizziness (Chai et al., 2022). The combination of cilostazol with other antiplatelet agents or its use as a monotherapy was associated with a higher incidence of general adverse events compared to the control group. Lee et al. (2010) found that the dropout rate was significantly higher and drug compliance lower in the cilostazol group than the placebo, possibly because of increased adverse effects such as headache. 6% of patients withdrew from the study because they could not tolerate headache or dizziness (Han et al., 2013). Within 14 days, headache developed in 5% in the dual group and in 1% in the aspirin group (P < 0.001), and others (mainly asymptomatic tachycardia) were seen in 3% in the dual group and 1% in the aspirin group (P = 0.009). However, these differences disappeared at 3 months: 6% versus 4% (P = 0.137) and 6% versus 5% (P = 0.437), respectively (Aoki et al., 2019). This coincides with the general reduction in adverse effects after 3 months in the present study. Whether the side effects of cilostazol in the early stage of treatment will be the key to stopping the drug is uncertain. However,in the CSPS study, the starting dose of cilostazol was provided at 100 mg qd, which was gradually increased to 100 mg bid over a period of 15 days (Hoshino et al., 2021), in order to prevent the occurrence of general adverse events (headache, tachycardia). Further studies are required to confirm whether this mode of dosing reduces the occurrence of general adverse events.

The interval between the onset of a stroke and the initiation of therapy was found to have a significant impact on clinical outcomes. In the short-term subgroup, patients received cilostazol within 2 weeks of the initial event. In contrast, the long-term group exhibited a larger time span (2 days–180 days) between the initiation of medication after the initial event and a non-uniform dose of medication, which may have been a confounding factor (Table 1). A meta-analysis that distinguished between long-term and short-term groups using a 12-month threshold revealed that stroke recurrence rates declined in both long-term and short-term cilostazol-treated patients. However, cerebrovascular events and intracranial hemorrhage exhibited a notable reduction exclusively in the long-term group (Tan et al., 2015). The difference in intracranial hemorrhage observed in the long-term cohort of this investigation was not statistically significant. This finding may be attributed to the disparity in grouping and the attenuation of cerebral vasospasm by cilostazol (Dayyani et al., 2022). A meta-analysis conducted in 2020 revealed that the majority of the observed benefit occurred in trials where patients were randomized at least 2 weeks after experiencing a stroke. Additionally, the analysis found that long-term treatment with cilostazol for a minimum duration of 6 months was not associated with an increased risk (McHutchison et al., 2020). In contrast, in another meta-analysis employing a 3-month cut-off, no significant difference was identified in the efficacy or safety of cilostazol between short-term and long-term administration (Tan et al., 2021). The aforementioned three studies did not analyze cilostazol in isolation or in conjunction with other agents. The present study analyzed cilostazol in combination with other agents in order to address some of its inherent limitations. Moreover, there was no considerable disparity in the prevalence of general adverse events in the short-term group with cilostazol combination therapy. Conversely, the incidence of general adverse events was diminished in the long-term group. It may be postulated that this outcome is attributable to body tolerance resulting from ethnicity, receptor downregulation (Douen et al., 2008), and alterations in pharmacokinetics. In this study, the recurrence of ischaemic stroke and any stroke was found to be reduced at a follow-up time greater than 3 months without an increase in the incidence of serious adverse events. Cilostazol-based DAPT is a promising long-term secondary stroke prevention option, similar to aspirin plus dipyridamole (Greving et al., 2019). This may be of clinical guidance.

The present study was conducted exclusively with Asian participants, which may limit the generalizability of its findings to other ethnic groups and dietary cultures. Secondly, it should be noted that not all of the included studies were randomized controlled trials (RCTs). One of the studies was a cohort study. The interval between the initial incident and the commencement of cilostazol-based DAPT exhibited considerable variability among the enrolled population. Due to the limited number of literature, subgroup analyses of acute- and chronic-phase IS were not conducted, and a more precise duration of DAPT could not be determined. Moreover, a paucity of data exists for the purpose of comparing the efficacy and safety of cilostazol with that of other antiplatelet agents, excluding aspirin and clopidogrel, and it is not possible to determine which antiplatelet drugs are better combined with cilostazol. In light of the significant morbidity and financial burden associated with recurrent stroke, it is imperative that larger trials be conducted to substantiate these findings.

## Conclusion

Dual antiplatelet therapy based on cilostazol has been demonstrated to significantly reduce the risk of ischemic stroke, recurrence of any stroke, and does not increase the incidence of serious, fatal events. Furthermore, the incidence of general adverse events has been shown to decrease with the duration of treatment. The efficacy and safety of cilostazol-based DAPT in the management of ischemic stroke have been demonstrated, particularly in the long-term period beyond 3 months, a finding that requires further corroboration through additional research.

## Data Availability

The original contributions presented in the study are included in the article/Supplementary Material, further inquiries can be directed to the corresponding authors.
