# Supplementary material for: New options for cilostazol-based dual antiplatelet therapy for ischaemic stroke prevention in East Asian populations: a systematic review and meta-analysis
Source: Front Pharmacol. 2025 Feb 19;16:1533674. doi: 10.3389/fphar.2025.1533674 (PMC11880014; doi:10.3389/fphar.2025.1533674)
Supplement: Supplementary file 1 [file DataSheet1.docx]

**Supplement**

**Table S1. Search Strategy**

| **Set** | **PubMed Query** | **Results** |
| --- | --- | --- |
| 1 | ("Ischemic Attack, Transient"[Mesh] or "TIA (Transient Ischemic Attack)" or "TIA, Brain" or "TIAs (Transient Ischemic Attack)" or "Transient Ischemic Attack" or "Attack, Transient Ischemic" or "Attacks, Transient Ischemic" or "Ischemic Attacks, Transient" or "Transient Ischemic Attacks" or "Cerebral Ischemia, Transient" or "Ischemia, Transient Cerebral" or "Transient Cerebral Ischemia" or "Transient Cerebral Ischemias") | 31669 |
| 2 | ("Ischemic Stroke"[Mesh] or "Stroke, Ischemic" or "Ischemic Strokes" or "Ischaemic Stroke*" or "Stroke, Ischaemic" or "Acute Ischemic Stroke*" or "Ischemic Stroke, Acute" or "Stroke, Acute Ischemic") | 44585 |
| 3 | ("Cilostazol"[Mesh] or "Pletal" or "OPC 13013" or "OPC-13013") | 1515 |
| 4 | #1 OR #2 | 73505 |
| 5 | #3 AND #4 | 103 |
|  |  |  |
| **Set** | **Cochrane Query** | **Results** |
| 1 | (Pletal):ti,ab,kw or (OPC 13013):ti,ab,kw or (OPC-13013):ti,ab,kw | 33 |
| 2 | MeSH descriptor: [Cilostazol] explode all trees | 448 |
| 3 | #1 OR #2 | 467 |
| 4 | MeSH descriptor: [lschemic Attack, Transient] explode all trees | 1141 |
| 5 | MeSH descriptor: [lschemic Stroke] explode all trees | 1522 |
| 6 | (Transient Ischemic Attack):ti,ab,kw or (TIA, Brain):ti,ab,kw or (TIA):ti,ab,kw or (Transient Ischemic Attack):ti,ab,kw or (Attack, Transient Ischemic):ti,ab,kw or (Attacks, Transient Ischemic):ti,ab,kw or (Ischemic Attacks, Transient):ti,ab,kw or (Transient Ischemic Attacks):ti,ab,kw or (Cerebral Ischemia, Transient):ti,ab,kw or (Ischemia, Transient Cerebral):ti,ab,kw or (Transient Cerebral Ischemia):ti,ab,kw or (Transient Cerebral Ischemias):ti,ab,kw or (Stroke, Ischemic):ti,ab,kw or (Ischemic Strokes):ti,ab,kw or (Ischaemic Stroke*):ti,ab,kw or (Stroke, Ischaemic):ti,ab,kw or (Acute Ischemic Stroke*):ti,ab,kw or (Ischemic Stroke, Acute):ti,ab,kw or (Stroke, Acute Ischemic):ti,ab,kw | 21766 |
| 7 | #4 OR #5 OR #6 | 21816 |
| 8 | #3 AND #7 | 91 |
|  |  |  |
| **Set** | **Embase Query** | **Results** |
| 1 | ischemic attack, transient'/exp OR 'ischemic attack, transient' OR 'tia'/exp OR 'tia' OR 'tia, brain' OR 'transient ischemic attack'/exp OR 'transient ischemic attack' OR 'attack, transient ischemic' OR 'attacks, transient ischemic' OR 'ischemic attacks, transient' OR 'transient ischemic attacks' OR 'cerebral ischemia, transient'/exp OR 'cerebral ischemia, transient' OR 'ischemia, transient cerebral' OR 'transient cerebral ischemia'/exp OR 'transient cerebral ischemia' OR 'transient cerebral ischemic' OR 'ischemic stroke'/exp OR 'ischemic stroke' OR 'ischemic strokes' OR 'stroke, ischemic' OR 'acute ischemic stroke*' OR 'ischemic stroke, acute' OR 'stroke, acute ischemic' | 191576 |
| 2 | Cilostazol' OR 'Pletal' OR 'OPC 13013' OR 'OPC-13013' | 7788 |
| 3 | #1 AND #2 | 961 |
|  |  |  |
|  |  |  |
| **Set** | **Medline Query** | **Results** |
| 1 | TS=(Cilostazol) OR TS=(Pletal) OR TS=(OPC 13013) OR TS=(OPC-13013) | 2241 |
| 2 | TS=(Ischemic Attack, Transient) OR TS=(TIA ) OR TS=(Transient Ischemic Attack) OR TS=(TIA, Brain) OR TS=(Transient Ischemic Attack) OR TS=(Attack, Transient Ischemic) OR TS=(Attacks, Transient Ischemic) OR TS=(Ischemic Attacks, Transient) OR TS=(Transient Ischemic Attacks) OR TS=(Cerebral Ischemia, Transient) OR TS=(Ischemia, Transient Cerebral) OR TS=(transient Cerebral Ischemia) OR TS=(Transient Cerebral Ischemic) | 42995 |
| 3 | TS=(Ischemic Stroke) OR TS=(Stroke, Ischemic) OR TS=(Ischemic Strokes) OR TS=(Stroke, Ischemic) OR TS=(Acute Ischemic Stroke*) OR TS=(Ischemic Stroke, Acute) OR TS=(Stroke, Acute Ischemic) | 107070 |
| 4 | #2 OR #3 | 129186 |
| 5 | #1 AND #4 | 262 |
|  |  |  |
|  |  |  |
| **Set** | **CNKI Query** | **Results** |
| 1 | 西洛他唑 | 750 |
| 2 | 缺血性脑卒中 + 缺血性脑梗死 + 短暂性脑缺血发作 + TIA | 72580 |
| 3 | #1 AND #2 | 46 |
|  |  |  |
| **Set** | **CBM Query** | **Results** |
| 1 | "缺血性脑卒中"[常用字段:智能] OR "缺血性脑梗死"[常用字段:智能] OR "短暂性脑缺血发作"[常用字段:智能] OR "TIA"[常用字段:智能] | 39084 |
| 2 | "西洛他唑"[常用字段:智能] | 943 |
| 3 | #1 AND #2 | 43 |
|  |  |  |
| **Set** | **VIP Query** | **Results** |
| 1 | 任意字段=缺血性脑卒中+缺血性脑梗死+短暂性脑缺血发作+TIA | 47863 |
| 2 | 任意字段=西洛他唑 | 985 |
| 3 | #1 AND #2 | 33 |
|  |  |  |
| **Set** | **Wangfang Query** | **Results** |
| 1 | 主题:(缺血性脑卒中) or 主题:(缺血性脑梗死) or 主题:(短暂性脑缺血发作) or 主题:(TIA) | 77620 |
| 2 | 主题:(西洛他唑) | 1250 |
| 3 | #1 AND #2 | 47 |

**Fig. S1. Bias analysis chart**

**
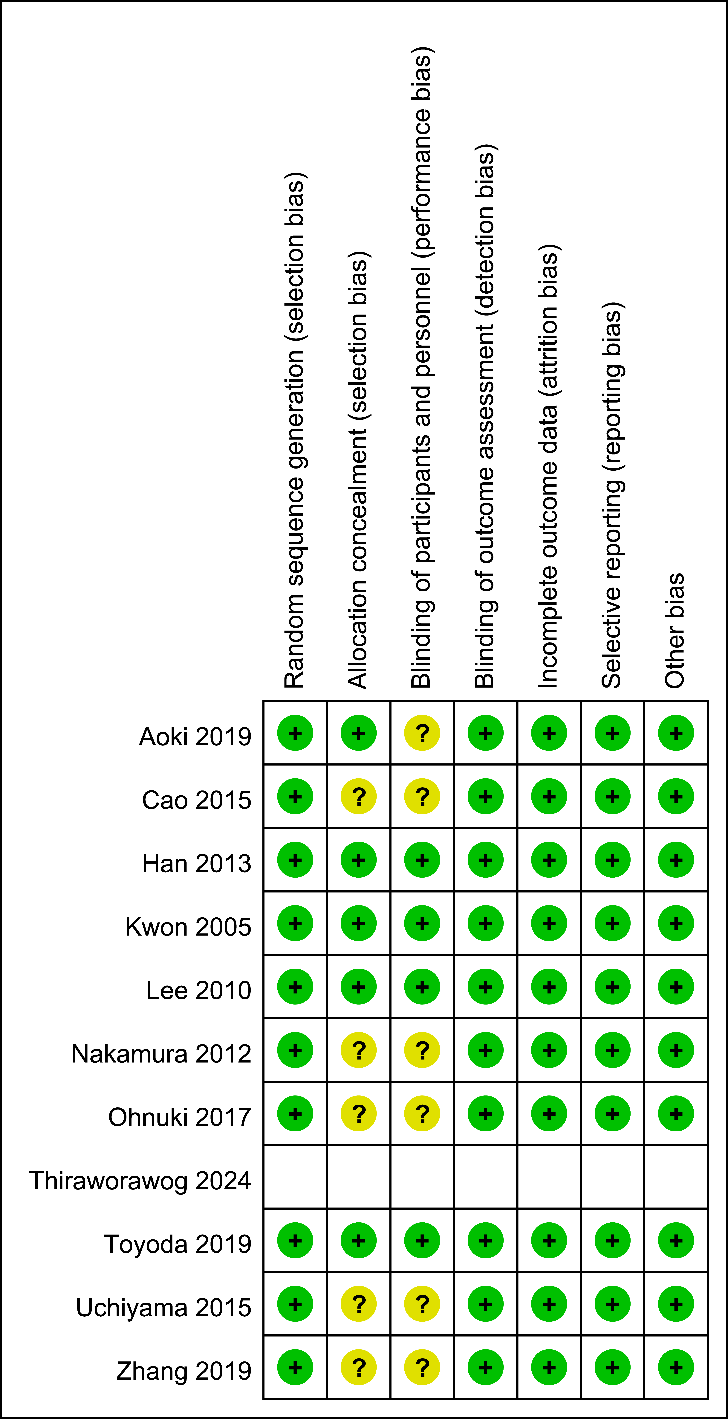
**

**Fig. S2. Sensitivity analysis of any stroke recurrence**

**
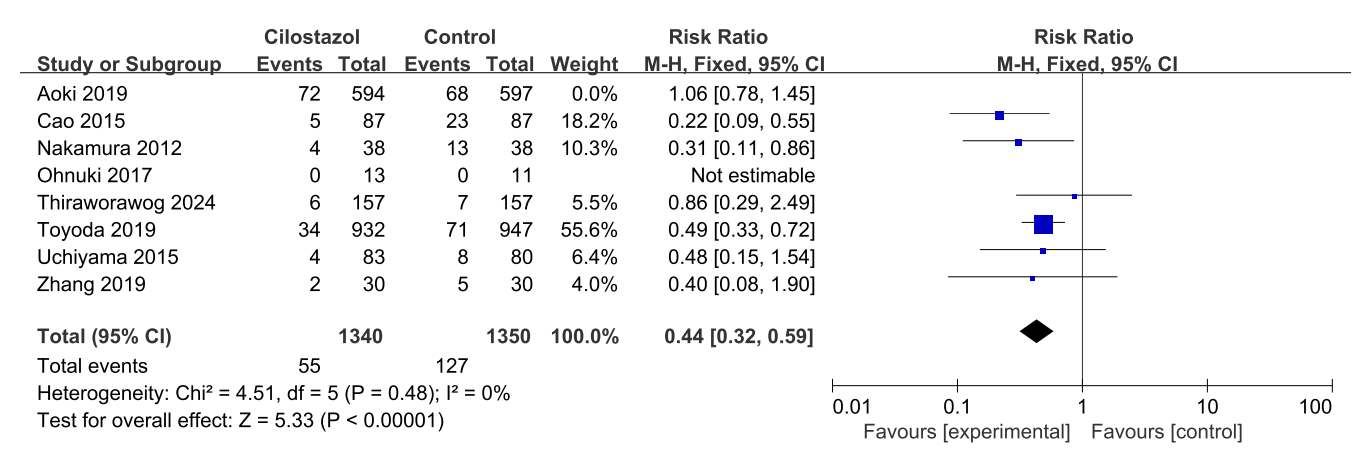
**

**Fig. S3. Sensitivity analysis of general adverse events**

**
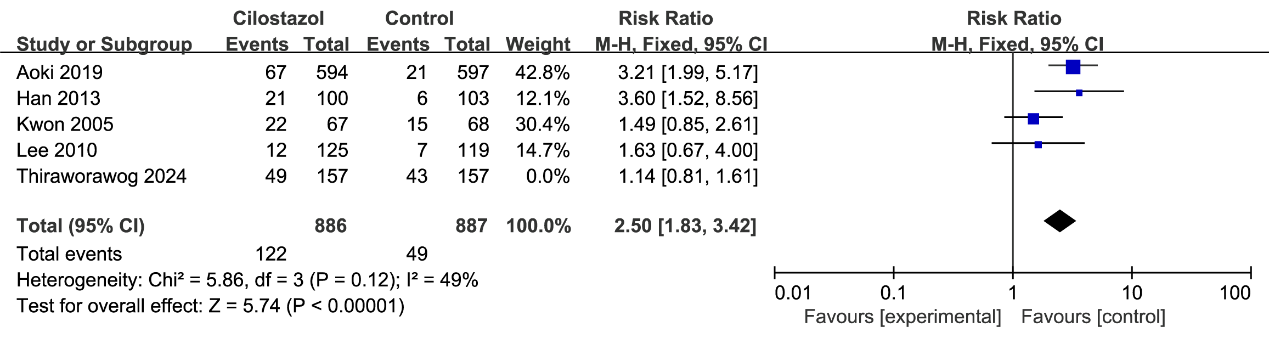
**

**Fig. S4. Subgroup analysis: ischemic stroke recurrence**

**
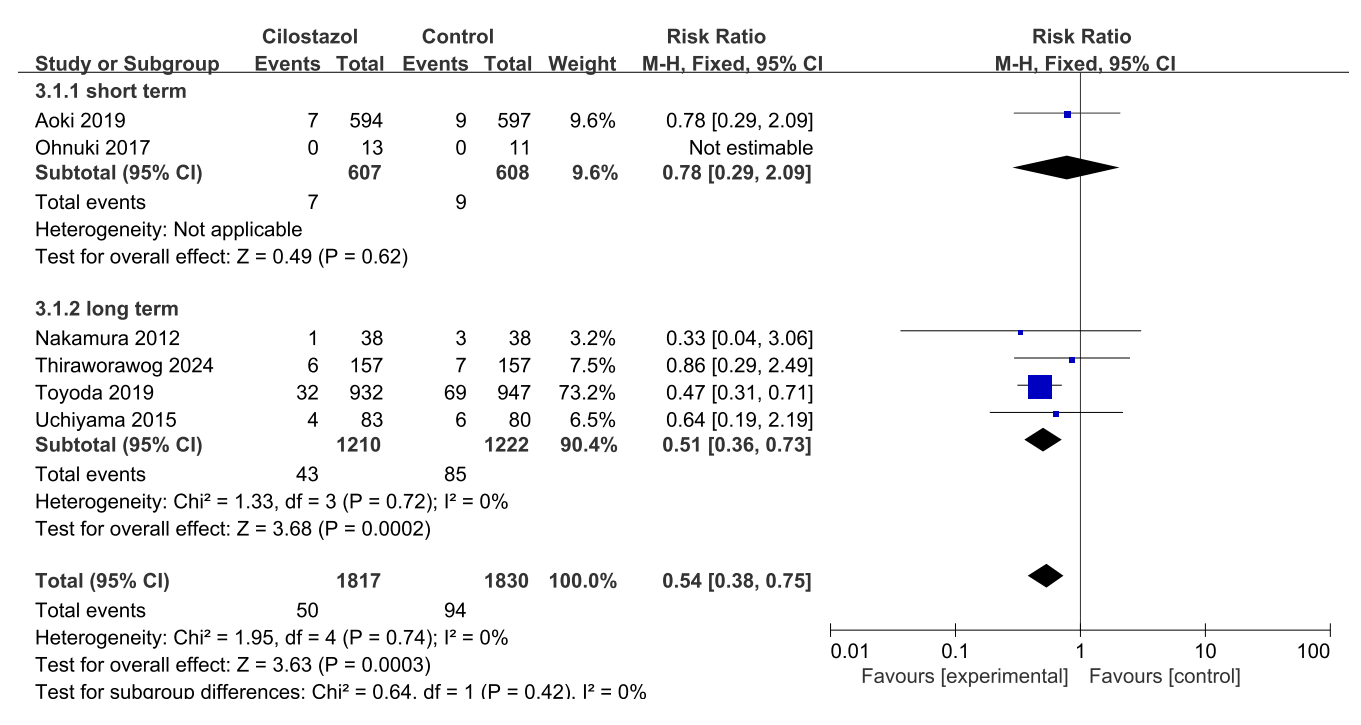
**

**Fig. S5A. Subgroup analysis: any stroke recurrence**

**
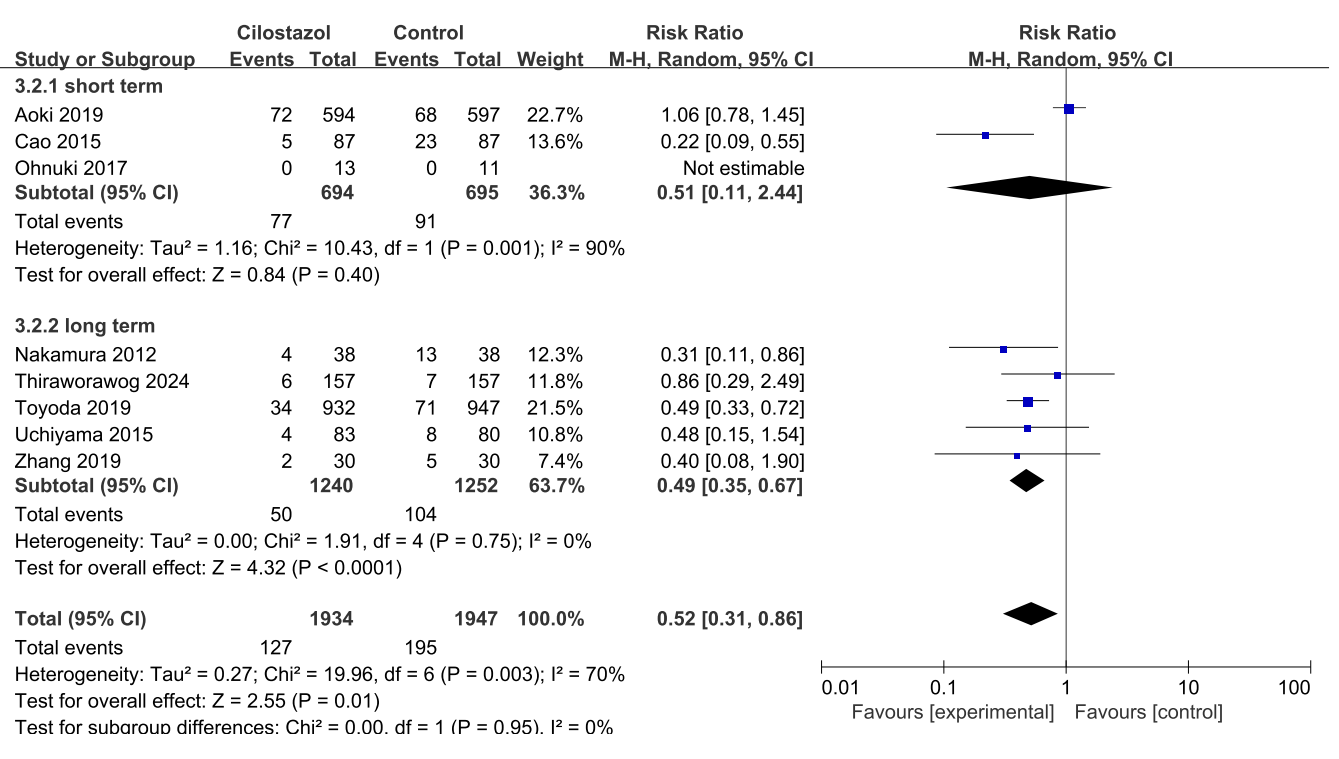
**

**Fig.S5B. Subgroup analysis: Sensitivity analysis of any stroke recurrence**

**
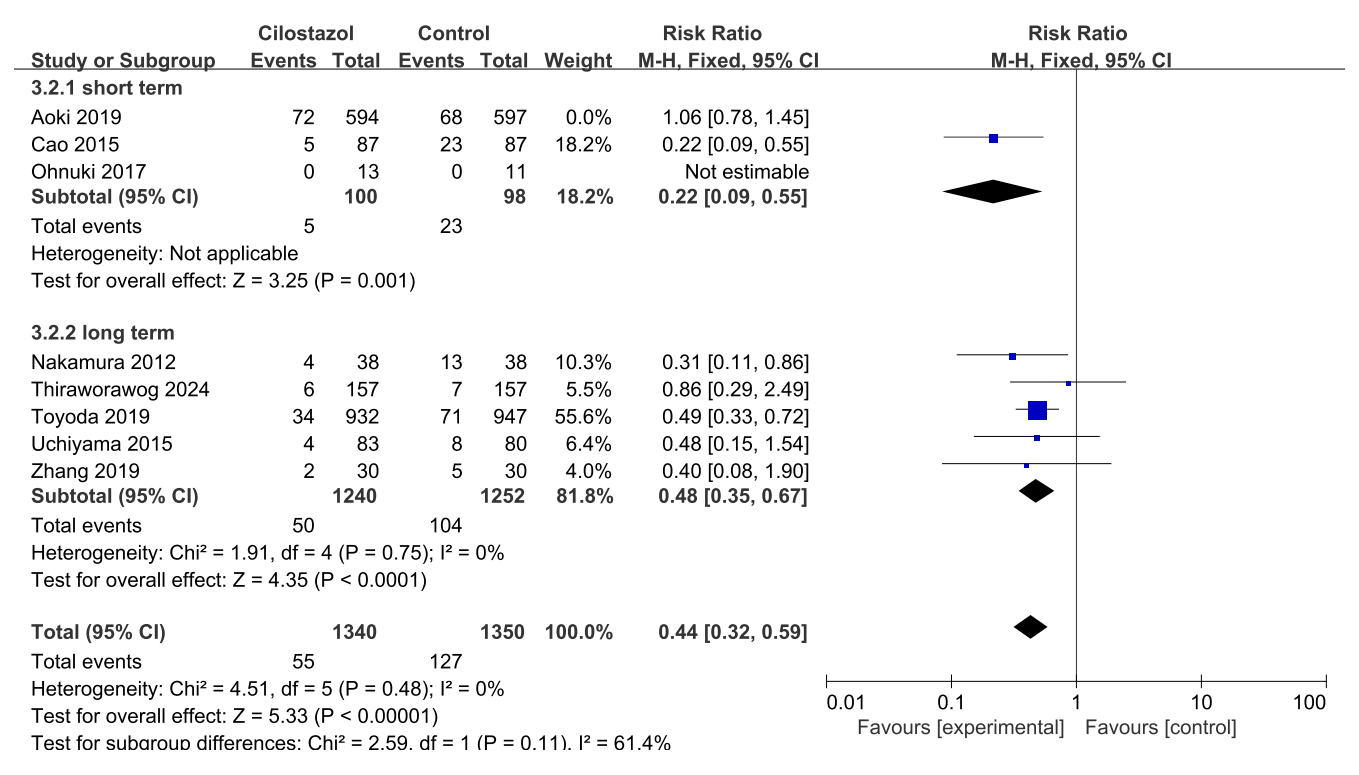
**

**Fig. S6. Subgroup analysis: intracranial hemorrhage**

**
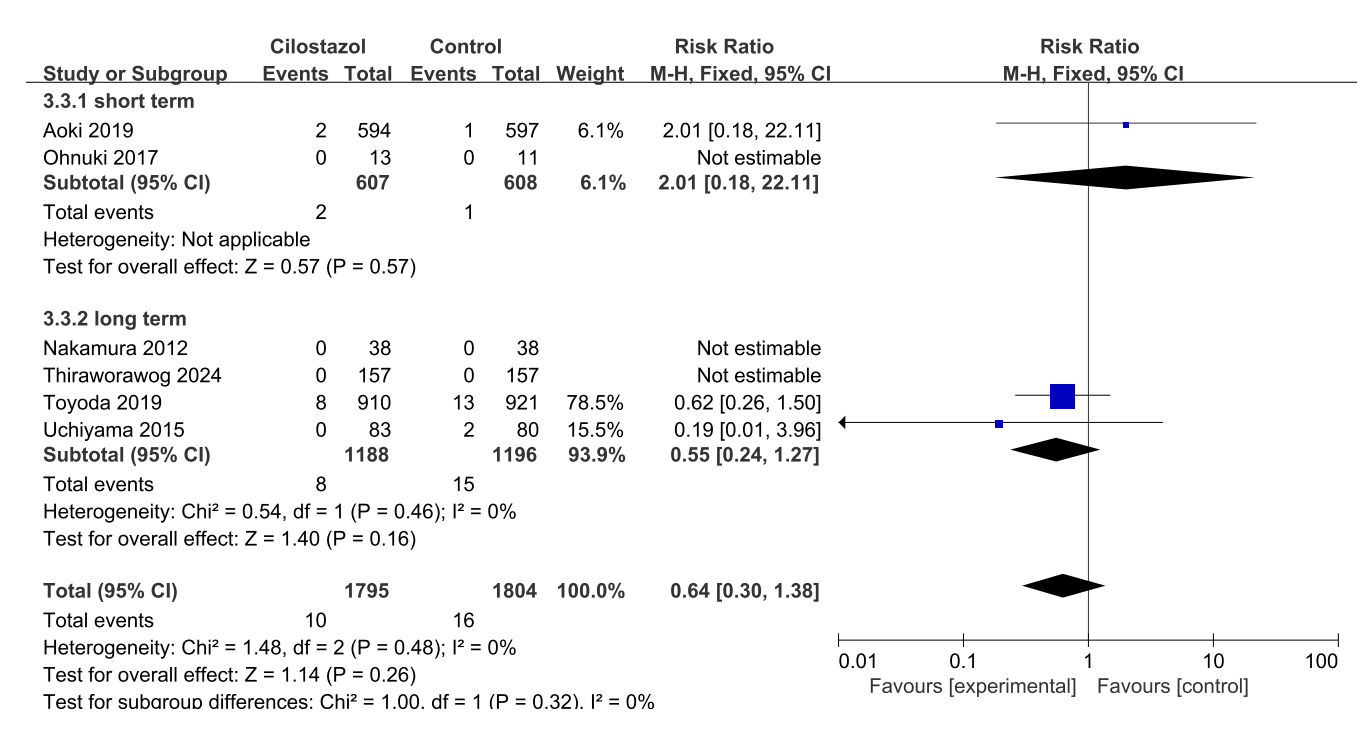
**

**Fig. S7. Subgroup analysis: cardiovascular events**

**
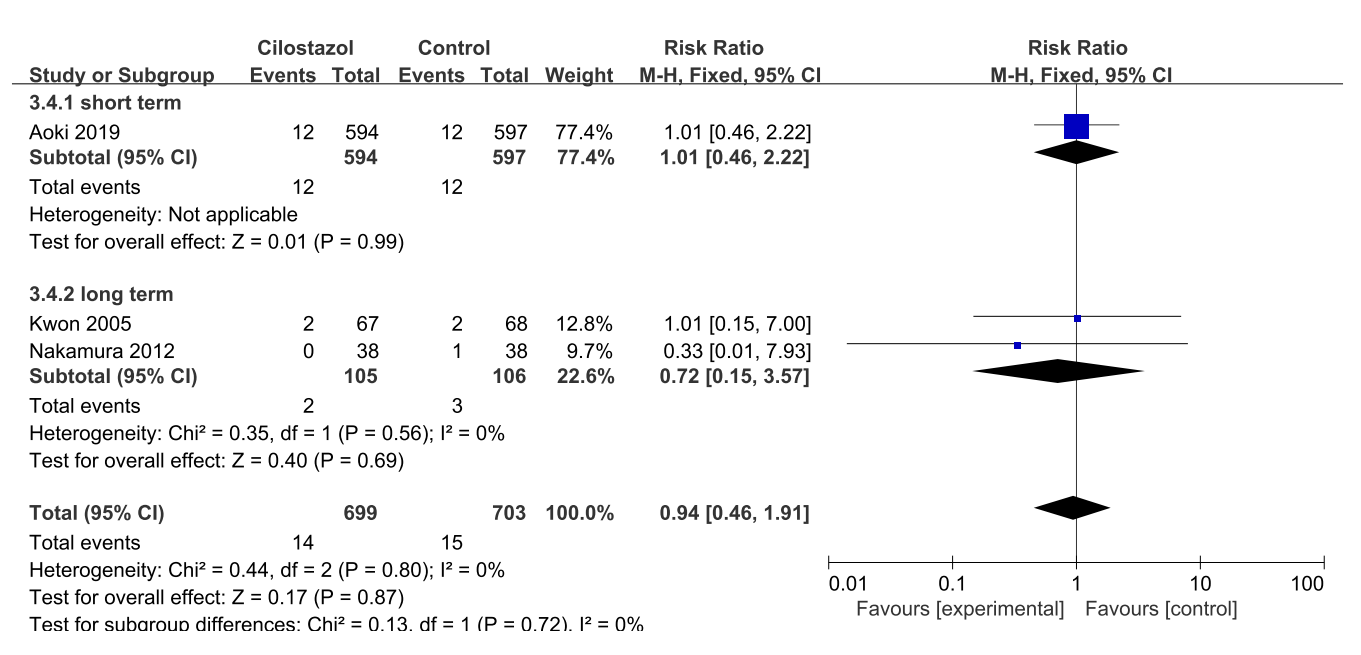
**

**Fig. S8. Subgroup analysis: bleeding events**

**
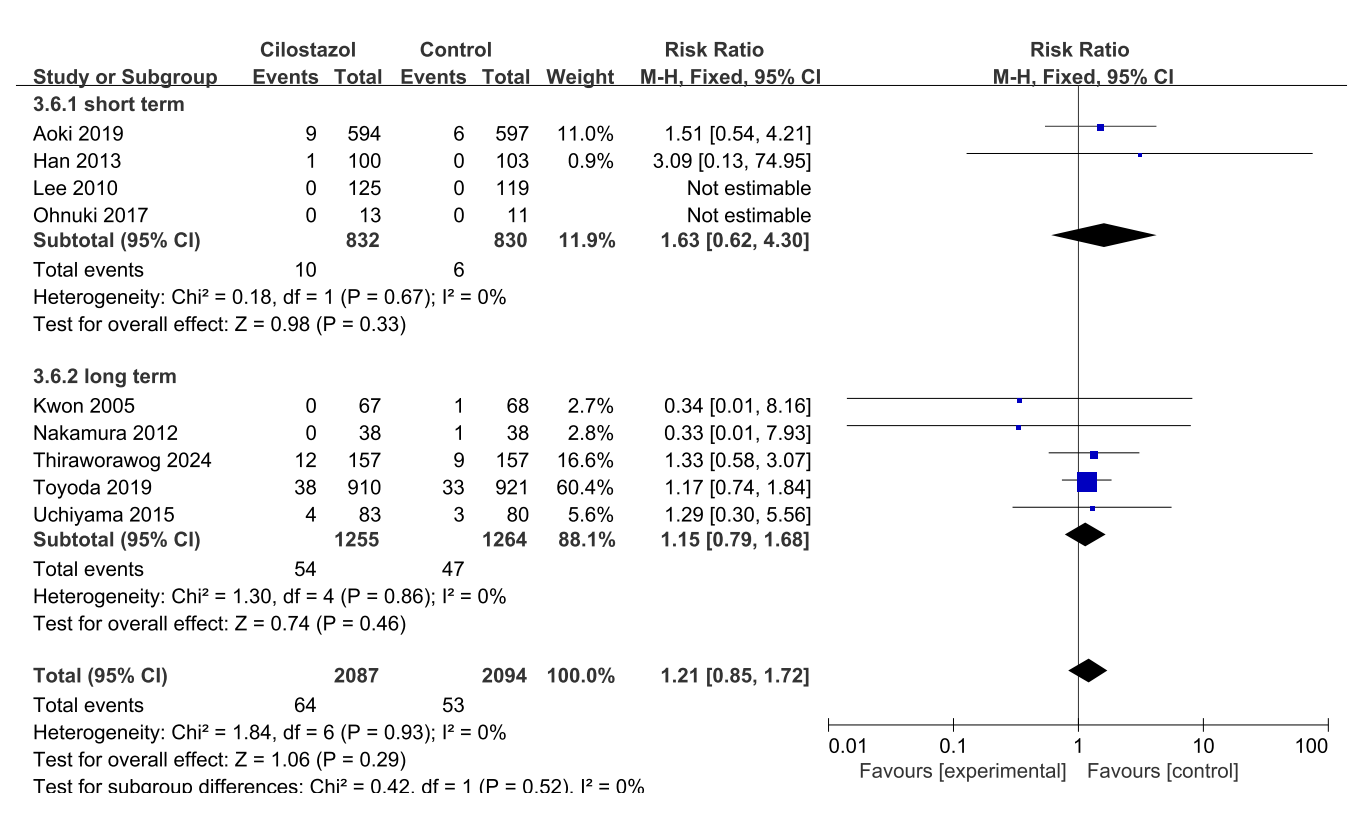
**

**Fig. S9A. Subgroup analysis: general adverse events**

**
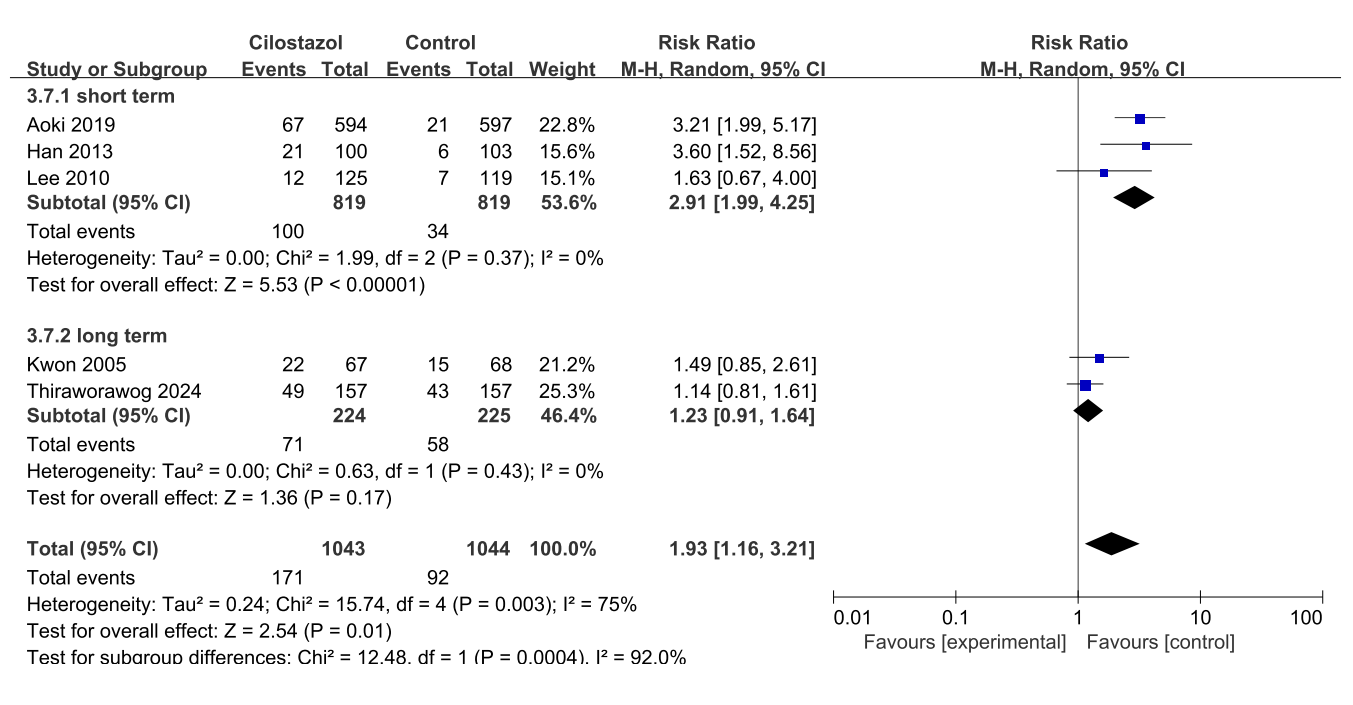
**

**Fig. S9B. Subgroup analysis: Sensitivity analysis of general adverse events**

**
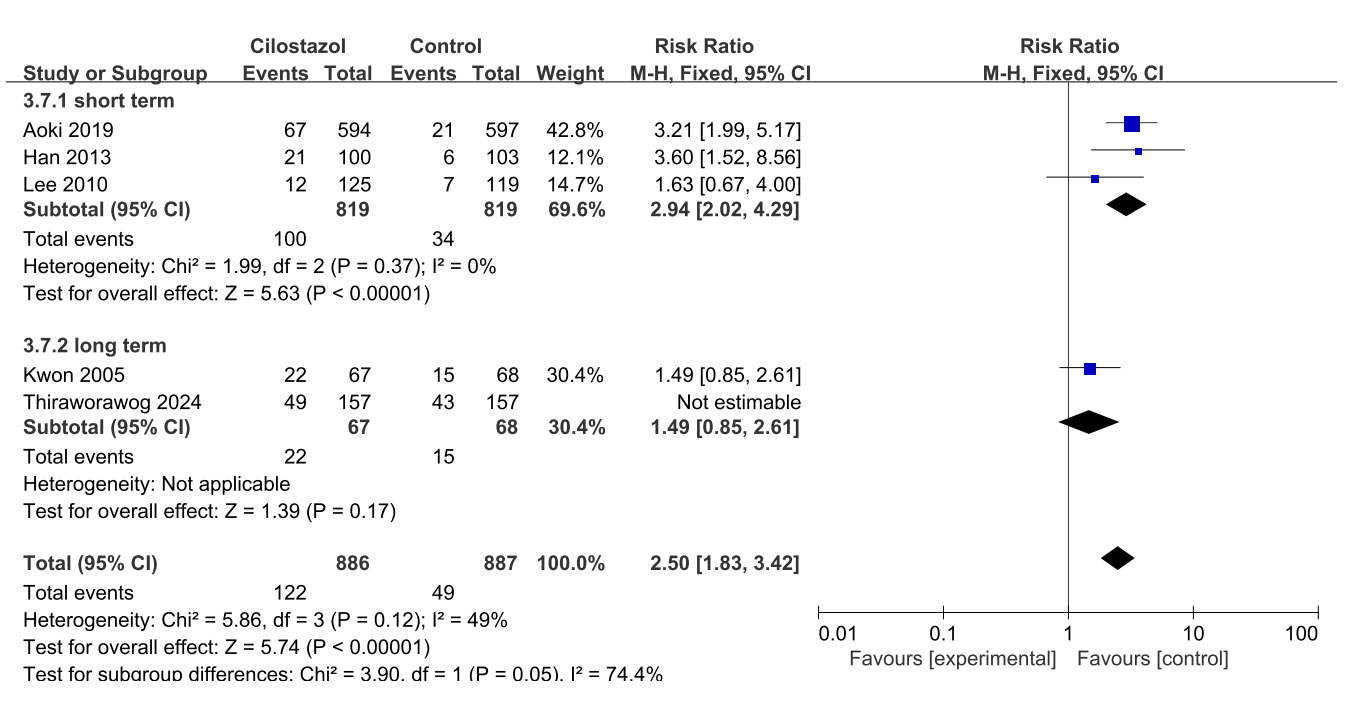
**
